# Reduction of Liftoff Effect in Eddy Current Measurement of Electrical Conductivity Using Multi-Frequency Excitation

**DOI:** 10.3390/s26020555

**Published:** 2026-01-14

**Authors:** Jiajie Wu, Yini Song, Shukai Chen, Yiru Xiao, Grzegorz Tytko, Yihua Kang, Bo Feng

**Affiliations:** 1 School of Mechanical Science and Engineering, Huazhong University of Science and Technology, Wuhan 430074, China; m202370901@hust.edu.cn (J.W.); songyini@hust.edu.cn (Y.S.); chenshukai7140@163.com (S.C.); yixiao@hust.edu.cn (Y.X.); yihuakang@hust.edu.cn (Y.K.); 2 Faculty of Automatic Control, Electronics and Computer Science, The Silesian University of Technology, Akademicka 16, 44-100 Gliwice, Poland; grzegorz.tytko@wp.pl

**Keywords:** eddy current testing, electrical conductivity, liftoff effect, multi-frequency, nondestructive testing

## Abstract

Eddy current testing is a widely used technique for electrical conductivity measurement due to its advantage of contactless measurement. However, the results are easily influenced by liftoff change. In this study, a new solution to compensate for the liftoff effect in eddy current measurements of conductivity is proposed. By measuring the inductance of coils with different frequencies, the liftoff–conductivity solution sets at each frequency are obtained from an analytical model. By finding a common solution for all frequencies, we can obtain the liftoff of the probe and the conductivity of the specimen simultaneously, thus improving the accuracy and reliability of conductivity measurements. For a liftoff variation of 1.14 mm, the introduced measurement error is up to 70.46% for aluminum alloys without liftoff compensation. By finding the common solution for multiple frequencies, the error is reduced to less than 5.57%. The selection of the frequency and tolerance limit is also discussed for the proposed method.

## 1. Introduction

Electrical conductivity is a critical parameter reflecting the electromagnetic properties of materials, directly influencing the performance and functionality of electrical equipment, transmission lines, and electrochemical devices. Therefore, the accurate measurement of material conductivity is of significant importance. Commonly used methods for measuring conductivity/resistivity include the four-point probe method and eddy current testing. The four-point probe method requires contact between the probe/electrode and the specimen [1,2,3,4], making it unsuitable for online automated measurement scenarios. In contrast, eddy current testing offers the advantage of non-contact measurement [5,6] and has broader application prospects in manufacturing.

In eddy current conductivity measurements, conductivity is typically reflected through coil impedance. Dodd and Deeds first derived the analytical solution for coil impedance [7], a model that calculates coil impedance based on given material properties. Conversely, if coil impedance is experimentally measured, material conductivity can also be estimated. Subsequent researchers simplified this analytical model, enabling the more efficient estimation of a specimen’s conductivity [8,9,10,11,12,13,14,15,16,17,18,19]. However, variations in the liftoff distance between the probe and the specimen during testing introduce significant signal interference, leading to substantial increases in conductivity measurement errors. This has become a core bottleneck limiting the online and automated application of eddy current testing. To suppress the liftoff effect, extensive research has been conducted by scholars worldwide.

Some researchers have designed specialized probe structures to reduce liftoff-induced signal interference at the hardware level, thereby avoiding complex post-processing. Bui et al. [20] employed a multichannel giant magnetoresistance probe, integrating the excitation coil as a displacement sensor to measure liftoff distance in real time. Duan et al. [21] developed a hybrid EMAT-PEC probe, using pulsed eddy current signals to synchronize the liftoff measurement and compensating for the amplitude of electromagnetic acoustic signals based on a model. Yin et al. [22] designed dual air-core coils and ferrite U-core coils, whose phase spectra remain unaffected by the liftoff distance under specific conditions. Lepage [23] introduced a flexible orthogonal array coil, where the ratio of signals between orthogonal and absolute channels cancels the amplitude impact of the liftoff on defect signals. Abu-Nabah et al. [24,25] revealed the competitive mechanism between inductive and capacitive liftoff effects. By optimizing probe design and calibrating with metal blocks of varying conductivities, they established a database. The resulting calibration model effectively reduces the sensitivity of high-frequency apparent eddy current conductivity spectroscopy measurements to liftoff effects. Ma et al. [26] integrated two traditional eddy current signal conditioning methods—bridge circuits and transformer-based induction—into a single probe. By leveraging the opposite impacts of liftoff on two output signals, they fused amplitude and phase information mathematically to cancel the liftoff interference.

Attempts have been made to eliminate liftoff interference through mathematical algorithms applied to raw signals. Fan et al. [27] performed frequency sweeps on specimens, corrected the model using multi-frequency data, and jointly inverted the liftoff distance and material conductivity. Ribeiro et al. [28] discovered that the liftoff effect attenuates high spatial frequency components of signals like a low-pass filter. During calibration, they derived a transfer function by analyzing spectral variations under different liftoff distances and applied deconvolution to partially compensate for the information loss caused by liftoff. Fu et al. [29] decomposed pulsed eddy current signals into multiple harmonics via fast Fourier transform, constructed dynamic trajectories for each harmonic, and identified defects while compensating for liftoff by analyzing the angular relationship between defect and liftoff trajectories. Some other researchers have proposed novel methods to reduce liftoff effects when measuring thickness and conductivity. Meng et al. [30] proposed an inversion algorithm based on the high-frequency sample-independent inductance characteristic. By leveraging the fact that inductance is sensitive to liftoff but insensitive to the parameters of non-magnetic materials at a specific high frequency, this method first retrieves the liftoff distance and then iteratively solves for material thickness using low-frequency signals, achieving a thickness measurement without calibration. This approach not only offers high computational efficiency but also simultaneously obtains both liftoff and thickness information. Huang et al. [31], on the other hand, introduced a liftoff variant feature in sweep-frequency eddy current testing. They designed a three-coil sensor and employed a twice differential signal processing method. By extracting the intersection points of sweep-frequency curves under different liftoff conditions—i.e., the characteristic frequency points insensitive to liftoff—they achieved the inversion of the material conductivity or thickness, effectively suppressing the influence of liftoff fluctuations.

In summary, existing liftoff compensation methods still face practical challenges, as some require prior knowledge of the material conductivity during the inversion process, and their anti-interference capability remains limited in complex industrial environments. On one hand, many approaches heavily rely on complex and time-consuming calibrations using standard specimens, where the calibration quality directly determines the compensation effectiveness. This limits their applicability in dynamic industrial environments. On the other hand, while some algorithms effectively suppress liftoff interference, their computational complexity prolongs the measurement time, making them unsuitable for high-speed, online detection.

This study proposes an eddy current conductivity measurement and liftoff compensation method based on multi-frequency excitation. By selecting multiple excitation frequencies to measure the sample and establishing conductivity–liftoff curves for each frequency, the material conductivity unaffected by liftoff is obtained by solving the intersection points. Furthermore, a tolerance mechanism is introduced, enabling the effective correction of results through the cross-validation of multi-frequency data even when the measurement results or parameter inversion for certain frequencies contain certain errors. This ensures a good detection accuracy in industrial field environments with strong interference. Additionally, this method requires only a single coil in the hardware, resulting in a simple structure and high reliability. In terms of functionality, it focuses on solving the problem of the accurate online measurement of conductivity under dynamic liftoff conditions, achieving a good balance between system complexity and industrial applicability. It provides an efficient and practical new solution for the application of eddy current testing technology in online sorting and real-time monitoring.

## 2. Measurement Principle and Analytical Model

### 2.1. Principle of Conductivity Detection

As a common non-destructive testing method, eddy current testing has been widely used in the inspection of various conductive materials. Based on the principle of electromagnetic induction, a single coil can be used to excite the eddy current and measure the conductivity. When an alternating current is passed through the coil, it generates an alternating magnetic field. As the coil approaches a metal specimen, eddy currents are induced within the metal. The induced secondary magnetic field from the eddy currents superimposes with the primary magnetic field of the coil, thereby affecting the complex impedance of the coil. The amplitude and phase of the eddy currents within the conductor are related to the physical properties of the metal being tested. In metals with different conductivities, the produced eddy currents and induced magnetic fields vary, allowing for the measurement of the metal conductivity by monitoring changes in the coil impedance.

### 2.2. Analytical Model

We assume that the measurement target is an infinitely large, homogeneous, and isotropic plane, with the coil parallel to the specimen and liftoff variations occurring only in the vertical direction. Additionally, edge effects and magnetic field distortions are neglected. Based on these assumptions, an analytical model of a single coil above the conductor is established. By deriving the coil’s impedance equation, the relationship between the coil’s inductance and the electrical conductivity of the test material can ultimately be obtained. This allows us to infer the conductivity of the material from the coil inductance measured in experiments. We let the coil have an inner radius *r_1_*, outer radius *r_2_*, liftoff height *z_1_*, and thickness *z_2_-z_1_*. The test specimen is a non-ferromagnetic metal with thickness *d*. According to Maxwell’s equations, the differential equation for the magnetic vector potential *A*(*r*,*z*) can be derived. In the cylindrical coordinate system, the governing equation is [7]

(1) $$\frac{\partial^{2}A}{\partial r^{2}}+\frac{1}{r}\frac{\partial A}{\partial r}+\frac{\partial^{2}A}{\partial z^{2}}-\frac{A}{r^{2}}=j\omega \mu \sigma A-\mu I_{0}-\mu \left[\frac{\partial \left(1/\mu \right)}{\partial r}\left(\frac{1}{r}\frac{\partial rA}{\partial r}\right)+\left(\frac{\partial \left(1/\mu \right)}{\partial z}\right)\frac{\partial A}{\partial z}\right]$$

where *A* is the magnetic vector potential, *ω* is the angular frequency of excitation, *μ* and *σ* are the magnetic permeability and conductivity of the sample, and *I*_0_ is the amplitude of excitation current.

**Figure 1:**
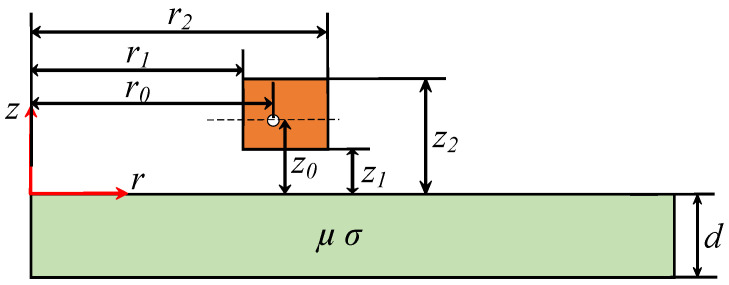
A schematic diagram of the analytical model.

To further understand the relationship between the coil impedance and the material property, the space is divided into four regions, three air regions (I, II, and IV) and one conductor region (III), as shown in Figure 1. Applying variable separation to magnetic vector potential *A*(*r,z*) = *R*(*r*)*Z*(*z*), the general solutions can be given in the following form [7]:

(2) $$A\left(r,z\right)=\int_{0}^{\infty }(CJ_{1}(\alpha r)+DY_{1}(\alpha r))\left(Ee^{\alpha_{i}z}+Fe^{-\alpha_{i}z}\right)d\alpha$$

where *C*, *D*, *E*, and *F* are coefficients to be determined; *J*_1_ (*αr*) and *Y*_1_ (*αr*) are the first-order Bessel function of the first and second kind; and *α* is the variable for integration.

Considering the boundary conditions, the coefficients in expressions can be determined, and the magnetic vector potential in regions I and II are obtained as follows:

(3) $$\begin{array}{cc} A^{I} & =\int_{0}^{\infty }\frac{I_{0}\mu_{0}r_{0}J_{1}(\alpha r_{0})J_{1}(\alpha r_{0})}{2K}\cdot [(\alpha_{1}^{2}\mu_{0}^{2}-\alpha^{2}\mu^{2})(1-e^{2\alpha_{1}d})\\ & +(2\alpha \alpha_{1}\mu \mu_{0}+\alpha^{2}\mu^{2}+\alpha_{1}^{2}\mu_{0}^{2})e^{2\alpha_{1}d+2\alpha z_{0}}+(2\alpha \alpha_{1}\mu \mu_{0}-\alpha^{2}\mu^{2}-\alpha_{1}^{2}\mu_{0}^{2})e^{2\alpha z_{0}}]e^{-\alpha z-\alpha z_{0}}d\alpha \end{array}$$

(4) $$A^{\mathrm{II}}=\int_{0}^{\infty }\frac{I_{0}\mu_{0}r_{0}J_{1}(\alpha r_{0})J_{1}(\alpha r_{0})}{2}\cdot [e^{\alpha z-\alpha z_{0}}-\frac{\left(\alpha^{2}\mu^{2}-\alpha_{1}^{2}\mu_{0}^{2})(e^{2\alpha_{1}d}-1\right)}{K}e^{-\alpha z-\alpha z_{0}}]d\alpha$$

where *μ*_0_ is the magnetic permeability of air.

For an axisymmetric *N* turn coil, its impedance *Z* is defined as the ratio of the induced voltage *V* to the excitation current *I*. This induced voltage arises directly from the variation in the total magnetic flux linkage of the coil, and the total flux linkage can be obtained by integrating the magnetic vector potential *A* over the entire cross-sectional area of the coil. The impedance of the final coil can be expressed as follows [32]:

(5) $$\begin{array}{l} Z=\frac{2j\cdot \omega \pi \mu_{0}N^{2}}{{\left(z_{2}-z_{1}\right)}^{2}{\left(r_{2}-r_{1}\right)}^{2}}\int_{0}^{\infty }\frac{I^{2}(r_{2},r_{1})}{\alpha^{6}}[\alpha (z_{2}-z_{1})+e^{\alpha (z_{1}-z_{2})}-1\\ +(e^{-2\alpha z_{2}}+e^{-2\alpha z_{1}}-2e^{-\alpha (z_{1}+z_{2})})\frac{\left(\alpha^{2}\mu^{2}-\alpha_{1}^{2}\mu_{0}^{2})(e^{2\alpha_{1}d}-1\right)}{2K}]d\alpha \end{array}$$

where $I\left(r_{2},r_{1}\right)\equiv \alpha^{2}\int_{r_{1}}^{r_{2}}r_{0}J_{1}\left(\alpha r_{0}\right)dr_{0}$ and $K\equiv (\alpha^{2}\mu^{2}+\alpha_{1}^{2}\mu_{0}^{2})(e^{2\alpha_{1}d}-1)+2\alpha \alpha_{1}\mu_{0}\mu (e^{2\alpha_{1}d}+1).$

Since the impedance of a coil is given by Z=R+jωL, the analytically derived coil inductance would be

(6) Lcoil=Im(Z)/ω

It can be seen from (5) and (6) that the coil inductance is a function of the conductivity of the test piece $L_{\mathrm{coil}}=f(\sigma )$. Under the condition of keeping other parameters constant, it can be concluded that the inductance of the coil is determined by the conductivity of the test piece. The steps to obtain the estimated conductivity are given as follows: (a) measure the inductance for a coil above the conductive specimen; (b) sweep the conductivity value and use (5) and (6) to calculate the corresponding analytical inductance values; and (c) choose the conductivity that outputs an inductance that matches the experimentally measured one to be the estimation of conductivity.

However, if the liftoff also changes during the measurement, the inductance is a function of both variables, which can be expressed as

(7) $$L_{\mathrm{coil}}=f(\sigma ,d)$$

In this case, the real-time liftoff value should be known to produce a precise estimation of the conductivity. However, the liftoff variation caused by the sample vibration is difficult to obtain. If a fixed liftoff value is used for the calculation, while the actual liftoff changes, errors will be presented in the estimated conductivity. The effect of liftoff changes on the conductivity measurement will be quantitatively analyzed in the next section.

## 3. Experiments

### 3.1. Experimental Platform

**Figure 2:**
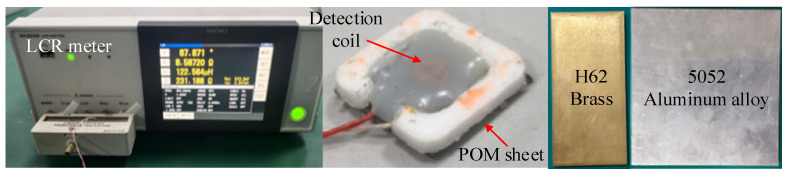
Materials and Instruments Used in the Experiment.

To verify the error bounds of the conventional single-frequency method under varying liftoff conditions and thereby validate the effectiveness of the multi-frequency intersection-based solution in mitigating the liftoff effect, an experimental platform was established as shown in Figure 2. The specimens to be tested are H62 brass and a 5052 aluminum alloy (Jiangsu Gefang New Materials Co., Ltd., Jingjiang, China), and their electrical conductivities are 14.08 × 10^6^ S/m and 20.65 × 10^6^ S/m, respectively. The dimension of the brass plate is 50 mm × 100 mm × 5 mm, and the dimension of the aluminum alloy plate is 100 mm × 100 mm × 5 mm. The detection coil has 163 turns, and the outer diameter, inner diameter, and height are 3.33 mm, 1.94 mm, and 1.01 mm, respectively. An LCR meter (Hioki IM3536, Hioki EE Corporation, Ueda City, Nagano Prefecture, Japan), with an accuracy of ±0.05%, is used to measure the inductance of the coil. Polyformaldehyde (POM) sheets of different thicknesses are inserted between the coil and the specimen to control the liftoff of the coil.

### 3.2. Estimation of Conductivity

**Figure 3:**
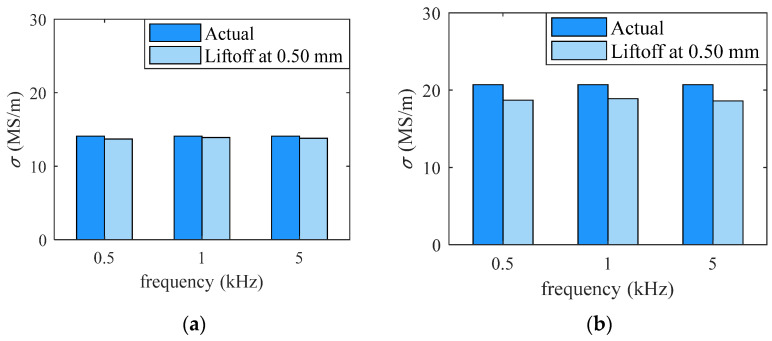
(**a**) Inductance value of brass obtained when liftoff is 0.5 mm; (**b**) inductance value of aluminum alloy obtained when liftoff is 0.5 mm.

In the first group of experiments, the liftoff between the detection coil and specimens was kept as 0.5 mm. The inductances of the coil were measured for different frequencies and materials. To estimate the conductivity, the known parameters, such as the coil dimension, liftoff, and plate thickness, are substituted into (5), then the conductivity is swept to calculate the inductance of the coil. The conductivity that outputs the inductance that matches the experimentally measured one would be the estimation of the conductivity of the specimen. The estimated conductivities for the brass and aluminum alloy specimens are provided in Table 1 and Figure 3. The largest relative error is 9.93%. This verifies that the eddy current testing method can be successfully used to measure the electrical conductivity of materials.

### 3.3. Liftoff Effects

In the process of automatic online measurements, there will be inevitable liftoff changes due to the vibration of the specimen. In order to produce an accurate estimation of conductivity, the instantaneous liftoff value should be known, so that the actual liftoff value can be used in (5) to calculate the coil inductance and compare it with the experimentally measured one. However, the instantaneous measurement of liftoff usually requires additional measurement devices, such as laser ranging devices. This is usually not possible due to the cost.

If the liftoff value is not instantaneously measured and used for the calculation of inductance, i.e., one fixed liftoff value is used in the analytical calculation even if the actual liftoff changes in the measurements, significant errors will be introduced in the conductivity estimation.

**Figure 4:**
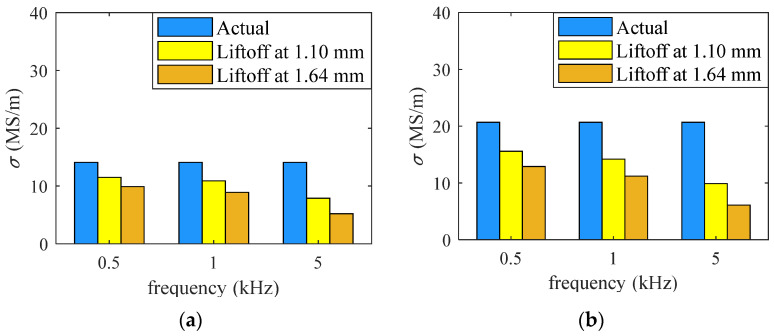
(**a**) Estimated conductivity of brass under the influence of liftoff change. (**b**) Estimated conductivity of aluminum alloy under the influence of liftoff change.

To test the influence of the liftoff change on the conductivity estimation, two additional groups of experiments were conducted, with the liftoff set to 1.10 mm and 1.64 mm. Coil inductances were measured at new liftoffs, while the original liftoff value of 0.50 mm was used to calculate the inductance using (5). In this case, the inversed conductivities contain large errors due to the incorrect liftoff values used in the analytical calculation. The results are shown in Table 2 and Figure 4. It can be seen that when the liftoff value is different from the one we set in (5), the estimated conductivities contain large errors. In the next section, a method of multi-frequency excitation will be used to reduce the error caused by the liftoff change in the measurement process.

## 4. Multi-Frequency Eddy Current Method

### 4.1. Multi-Frequency Signal Processing Procedures

According to (5), there will be different combinations of conductivity and liftoff that produce the same inductance output. Thus, with an experimentally measured coil inductance, it is not possible to estimate the conductivity and liftoff simultaneously. A solution proposed in this study is to use multi-frequency excitation in the eddy current testing. For one frequency, there will be a solution set for the conductivity and liftoff (a series of conductivity liftoff pairs) that fit the measured inductance. With one frequency, it is not possible to find the correct pair, since all the pairs make the calculated inductance match the experimentally measured one. However, if more frequencies are considered, each frequency would provide a solution set for the conductivity and liftoff. The common conductivity liftoff pair that appears in all the frequencies would be the actual conductivity and liftoff, because they make the results at all frequencies match with the experimentally measured ones.

**Figure 5:**
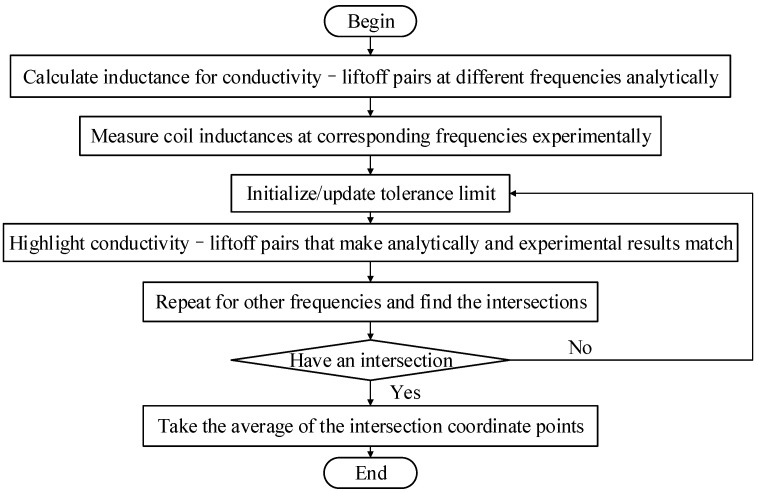
Flow chart of multi-frequency eddy current testing method.

The flowchart for the processing procedures is presented in Figure 5. Firstly, the coil inductances for all conductivity–liftoff pairs within a certain range are calculated at different frequencies according to (5) and (6) to construct an inductance dataset. Then, experiments are made to measure the inductance of the coil above a conductive plate at the corresponding frequencies. Thirdly, a tolerance limit is set, which is the allowed differences between experimentally measured and analytically calculated inductances. Fourthly, to visualize the process, a contour map is plotted as the coordinate set, with the liftoff being the abscissa and the conductivity being the ordinate. The conductivity–liftoff pairs that cause the difference between analytically calculated and experimentally measured inductances within the tolerance limit are highlighted in the contour map for one frequency. Then, the process is repeated for other frequencies, and the intersection of highlighted conductivity–liftoff pairs at different frequencies would be the estimation of the conductivity and liftoff. If no intersection is found, the tolerance limit should be increased; if the intersection region is too large, the tolerance limit should be decreased. This adjustment process is repeated until an appropriate intersection region is obtained. The final results for the liftoff distance and conductivity are obtained by summing the horizontal and vertical coordinates of all points within the intersection area, respectively, and calculating their averages.

### 4.2. Multi-Frequency Processing Results

**Figure 6:**
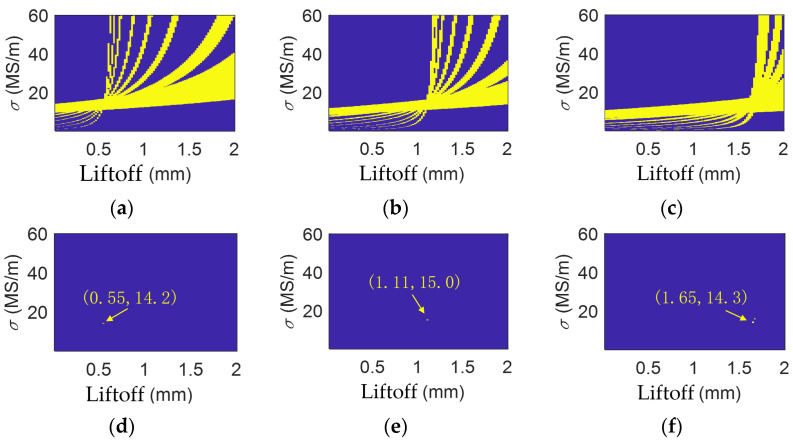
Multi-frequency data of brass when liftoff is (**a**) 0.50 mm; (**b**) 1.10 mm; and (**c**) 1.64 mm. Multi-frequency processing results for brass when liftoff is (**d**) 0.50 mm; (**e**) 1.10 mm; and (**f**) 1.64 mm.

The analytical calculations of impedances were performed in MATLAB R2018a. The inductance datasets were constructed by setting the liftoff range to 0.02 mm to, 2.00 mm, with a step of 0.02 mm, and the conductivity range to 1 × 10^5^ S/m to 6 × 10^7^ S/m, with a step of 1 × 10^5^ S/m. The variation range of the liftoff is defined based on actual inspection conditions, and the vertical axis range is determined according to the expected conductivity variation in the test material. The step sizes along both horizontal (liftoff) and vertical (conductivity) axes must balance the efficiency of constructing the inductance dataset with measurement accuracy: an excessively small step size significantly increases the time required to build the dataset, while an overly large step size leads to greater experimental errors. Therefore, appropriate step sizes should be selected according to the precision requirements of the specific application. For instance, when the liftoff step size is set to 0.02 mm, the corresponding liftoff detection error is ±0.02 mm. The datasets of coil inductances under different liftoffs and conductivities are calculated at the frequencies of 1 kHz, 2 kHz, 5 kHz, 10 kHz, 20 kHz, 50 kHz, 100 kHz, and 300 kHz. Coil inductances are also experimentally measured at these frequencies at liftoffs of 0.50 mm, 1.10 mm, and 1.64 mm. The tolerance limit is set to ±0.2%. At one frequency, the conductivity–liftoff pairs that output an analytically calculated inductance that is 1 ± 0.2% of the experimentally measured one are highlighted. This process was repeated for other frequencies, and the results are shown in Figure 6.

In Figure 6, the figures on the left-hand side show the highlighted regions for all the frequencies, and the figures on the right-hand side show the intersections of the highlighted regions. The coordinate of this intersection point gives the estimated coil liftoff and the conductivity of the brass. As shown in Figure 6, the multi-frequency processing liftoff results produce an estimated conductivity of 14.2 × 10^6^ S/m, 15.0 × 10^6^ S/m, and 14.3 × 10^6^ S/m at liftoffs of 0.50 mm, 1.10 mm, and 1.64 mm. Their relative errors are 0.85%, 6.53%, and 1.56%.

**Figure 7:**
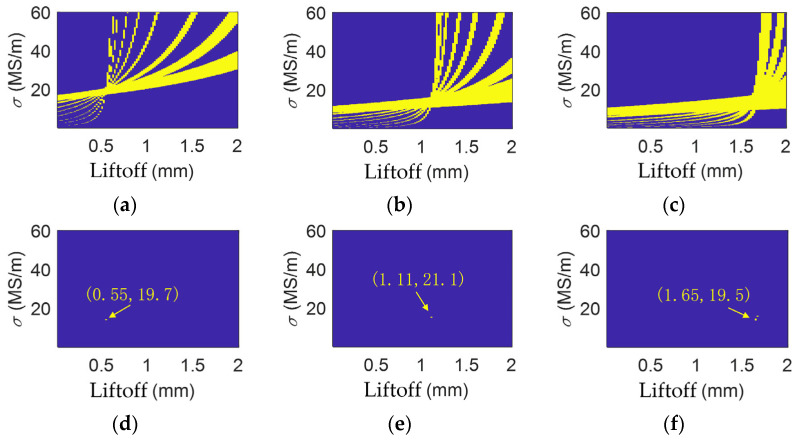
Multi-frequency data of aluminum when liftoff is (**a**) 0.50 mm; (**b**) 1.10 mm; and (**c**) 1.64 mm. Multi-frequency processing results for aluminum when liftoff is (**d**) 0.50 mm; (**e**) 1.10 mm; and (**f**) 1.64 mm.

The multi-frequency processing results for the aluminum alloy are shown in Figure 7. At different liftoffs, with tolerance limits set to 0.13%, 0.18%, and 0.175%, the obtained electrical conductivities of the aluminum alloy are 19.7 × 10^6^ S/m, 21.1 × 10^6^ S/m, and 19.5 × 10^6^ S/m at liftoffs of 0.50 mm, 1.10 mm, and 1.64 mm, and their relative errors are 4.60%, 2.18%, and 5.57%.

According to the experimental results, it can be seen that the conductivity of materials can be measured accurately by the multi-frequency measurement method. When the liftoff changes, this method can not only effectively eliminate the influence of liftoff on the experiment but also obtain the actual liftoff values.

### 4.3. The Influence of the Frequency Selection on the Results

**Figure 8:**
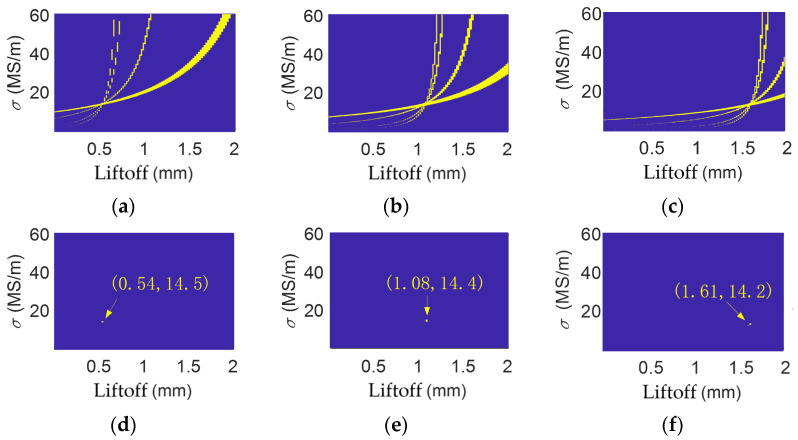
Data for 2 kHz, 10 kHz, 50 kHz, and 100 kHz for the brass specimen when liftoff is (**a**) 0.50 mm; (**b**) 1.10 mm; and (**c**) 1.64 mm. Common solution for brass when liftoff is (**d**) 0.50 mm; (**e**) 1.10 mm; and (**f**) 1.64 mm.

**Figure 9:**
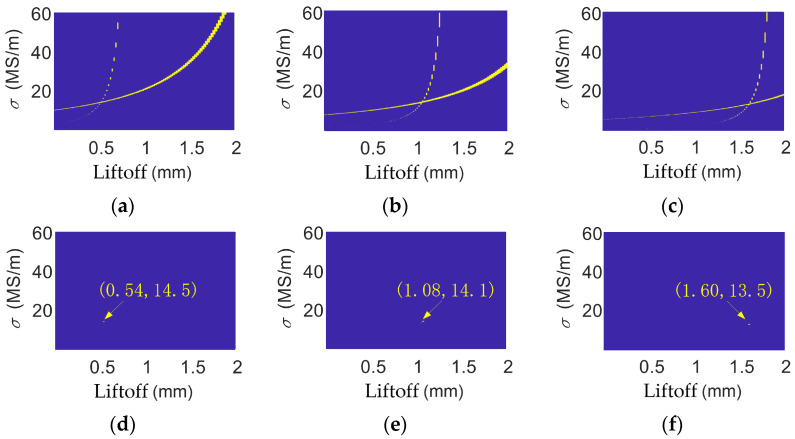
Data for 2 kHz and 50 kHz for the brass specimen when liftoff is (**a**) 0.50 mm; (**b**) 1.10 mm; and (**c**) 1.64 mm. Common solution for brass when liftoff is (**d**) 0.50 mm; (**e**) 1.10 mm; and (**f**) 1.64 mm.

When using the multi-frequency detection method to measure conductivity, the frequency selected will affect the accuracy of the results. As shown in Figure 8, when the detection frequencies are only 2 kHz, 10 kHz, 50 kHz, and 100 kHz, with the tolerance limits set to 0.12%, 0.12%, and 0.07%, the conductivities obtained for brass are 1.45 × 10^7^ S/m, 1.44 × 10^7^ S/m, and 1.42 × 10^7^ S/m, respectively, in different liftoff conditions. The relative errors are 2.84%, 2.13%, and 0.71%. If only two frequencies, 2 kHz and 50 kHz, are selected, as shown in Figure 9, with the tolerance limits set as 0.06%, 0.06%, and 0.03%, the conductivities obtained are 1.45 × 10^7^ S/m, 1.41 × 10^7^ S/m, and 1.35 × 10^7^ S/m, respectively, in different liftoff conditions. The relative errors are 2.84%, 0%, and 4.26%.

From the above experimental results, it can be observed that when using multi-frequency eddy current testing, the selection of frequencies is crucial. If too few frequencies are chosen, a single measurement error can more easily affect the calculated conductivity. The more frequencies selected, the larger the tolerance required to ensure the intersection of multiple frequencies. Consequently, there is a smaller impact of single measurement errors on the results and a better stability of the results. However, this will demand more experimental time.

### 4.4. The Influence of the Tolerance Limit

**Figure 10:**
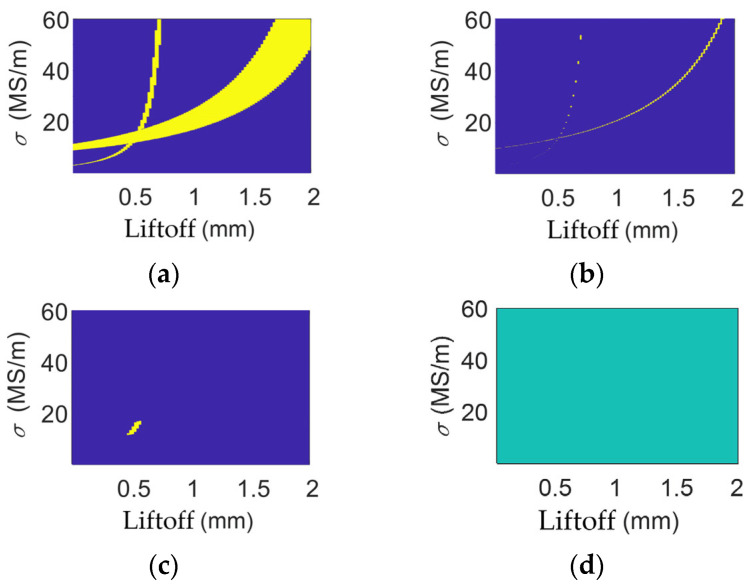
(**a**) Data for 2 kHz and 50 kHz with 0.5% tolerance limit. (**b**) Data for 2 kHz and 50 kHz with 0.03% tolerance limit. (**c**) Intersected region with 0.5% tolerance limit. (**d**) Intersected region with 0.03% tolerance limit.

When using the multi-frequency eddy current method to detect the conductivity of materials, the selection of the tolerance limit will affect the number of liftoff–conductivity pairs at each frequency. As shown in Figure 10, we choose excitation frequencies of 2 kHz and 50 kHz to measure the conductivity of brass and to achieve the common solution at the two frequencies. When the tolerance limit is large (0.5%), their intersection area is too large, which leads to a relatively significant error in the calculated conductivity. When the tolerance limit is too small, there may be no common solution. Therefore, in the flowchart shown in Figure 5, there should be a step to update the tolerance limit. We start with a small tolerance limit value, and if there is no common solution (i.e., intersected region), the tolerance limit will be increased until a common solution is found.

### 4.5. Thin Specimen Testing

**Figure 11:**
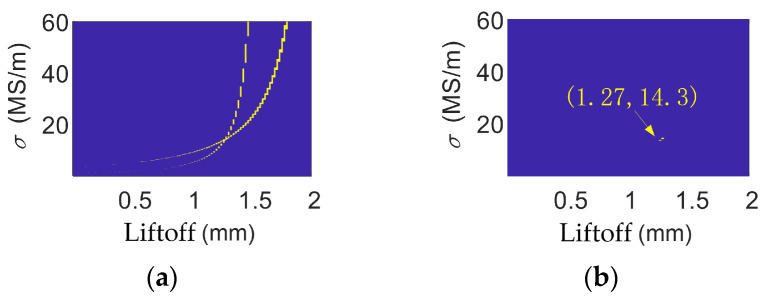
(**a**) Data for 10 kHz and 50 kHz for the brass specimen when liftoff is 1.30 mm. (**b**) Common solution for brass when liftoff is 1.30 mm.

To verify the effectiveness of the proposed method in thin specimen inspection scenarios, a brass thin specimen with dimensions of 50 mm × 100 mm × 1.12 mm was employed in experimental validation conducted at a liftoff distance of 1.3 mm. The experimental results are shown in Figure 11. When the excitation frequencies were set to 10 kHz and 50 kHz, respectively, and the tolerance threshold was set to 0.07, the measured electrical conductivity of the brass specimen was 1.43 × 10^7^ S/m, yielding a relative error of only 1.42%. These results demonstrate that the proposed method still achieves an effective liftoff compensation even for thin specimens. It should be noted, however, that when measuring thin specimens, the selected inspection frequency must satisfy the condition that the skin depth at that frequency is less than the specimen thickness, in order to avoid introducing additional measurement errors.

### 4.6. Parameter Selection

When using the multi-frequency intersection-based solution described in this paper to solve for the electrical conductivity of materials, in order to balance both detection accuracy and efficiency, it is necessary to comprehensively consider a parameter selection based on the detection principle and actual requirements. The specific optimization suggestions are as follows:

The selection of excitation frequencies must satisfy dual conditions: We should ensure that the skin depth at the chosen frequency is less than the specimen thickness, preventing a decline in detection accuracy due to excessive signal penetration. We should maximize the interval between excitation frequencies—this will result in more distinct differences in the slopes of the conductivity–liftoff curves corresponding to different frequencies, thereby creating a smaller intersection area and effectively reducing measurement errors.

The horizontal and vertical coordinate ranges of the dataset should be determined based on the actual detection scenario: The horizontal coordinate (liftoff distance) range should cover the possible variations in liftoff during the detection process. The vertical coordinate (conductivity) range should match the expected conductivity fluctuation range of the object under test, ensuring the completeness and applicability of the dataset.

The selection of step sizes for the horizontal and vertical coordinates must balance detection accuracy and efficiency: Appropriate step sizes should be set according to the actual detection accuracy requirements. This ensures that overly large step sizes do not lead to data discretization and compromise the detection accuracy, while overly small step sizes do not excessively prolong the database construction time, achieving a balance between precision and efficiency.

The selection of the tolerance value should be dynamically adjusted according to the size of the intersection area: A tolerance value suitable for the scale of the intersection should be selected. Additionally, by calculating the arithmetic mean of all coordinate values within the intersection, random errors can be further mitigated, enhancing the stability of the detection results.

## 5. Conclusions

In this article, the influence of the liftoff variation on the eddy current measurement of electrical conductivity was studied. The results showed that, if the liftoff value was correctly set to 0.5 mm in the analytical equation, the estimated conductivities for the H62 brass and 5052 aluminum alloy are less than 2.70% and 9.93%, respectively. If the liftoff varied to 1.64 mm, while 0.5 mm was still used in the analytical calculation, errors up to 63.07% and 70.46% could be introduced for the H62 brass and 5052 aluminum alloy.

To overcome this problem, a multi-frequency eddy current testing method and corresponding signal processing scheme were proposed. For each frequency, a conductivity–liftoff solution set was obtained, and the common solutions for multiple frequencies are chosen to be the estimated conductivity and liftoff. With the proposed method, the errors for the conductivity measurement were reduced to 6.53% and 5.57% for the brass and aluminum alloy under the liftoff variation from 0.5 mm to 1.64 mm.

## Figures and Tables

**Table 1:** Estimation of conductivity under correctly set liftoff value.

Excitation Frequency	Material	Inductance (μH)	Estimated Conductivity (MS/m)	Actual Conductivity (MS/m)	Relative Error
500 Hz	Brass	121.39	13.7	14.1	2.70%
	Aluminum alloy	120.92	18.7	20.7	9.44%
1 kHz	Brass	120.09	13.9	14.1	1.28%
	Aluminum alloy	119.26	18.9	20.7	8.47%
5 kHz	Brass	113.77	13.8	14.1	1.99%
	Aluminum alloy	112.06	18.6	20.7	9.93%

**Table 2:** Estimation of conductivity under incorrectly set liftoff value.

Excitation Frequency	Liftoff (mm)	Material	Inductance (μH)	Estimated Conductivity (MS/m)	Actual Conductivity (MS/m)	Relative Error
500 Hz	1.10	Brass	121.59	11.5	14.1	18.32%
		Aluminum alloy	121.21	15.6	20.7	24.46%
	1.64	Brass	121.74	9.9	14.1	29.69%
		Aluminum alloy	121.46	12.9	20.7	37.53%
1 kHz	1.10	Brass	120.63	10.9	14.1	22.59%
		Aluminum alloy	120.03	14.2	20.7	31.23%
	1.64	Brass	121.01	8.9	14.1	36.79%
		Aluminum alloy	120.57	11.2	20.7	45.76%
5 kHz	1.10	Brass	116.51	7.9	14.1	43.89%
		Aluminum alloy	115.47	9.9	20.7	52.06%
	1.64	Brass	118.22	5.2	14.1	63.07%
		Aluminum alloy	117.58	6.1	20.7	70.46%

## Data Availability

The original contributions presented in this study are included in the article. Further inquiries can be directed to the corresponding author.
